# Reconstruction of tibial bone defect in new age using the old age Papineau technique: A case series

**DOI:** 10.1016/j.amsu.2019.10.028

**Published:** 2019-11-05

**Authors:** Bambang Gunawan, Mohammad Triadi Wijaya, Muhammad Alvin Shiddieqy Pohan

**Affiliations:** a Department of Orthopaedics and Traumatology, Universitas Indonesia, Cipto Mangunkusumo Hospital, Jakarta, Indonesia; b Resident of Orthopaedic Surgery, Universitas Indonesia, Cipto Mangunkusumo Hospital, Jakarta, Indonesia

**Keywords:** Papineau, Bone defect, Tibia, Chronic osteomyelitis

## Abstract

**Introduction:**

Despite advances in treatment, chronic osteomyelitis and infected nonunion after trauma remain a challenge to the orthopaedic field. The Papineau technique, firstly described in 1973, is an alternative to treat such conditions in hospitals where microsurgery is not available, making closure of defect using flap is not feasible. We described our experience in treating patients with chronic osteomyelitis and infected non-union of tibial fractures using the Papineau technique.

**Methods:**

We reviewed the records of patients with severe open tibial fractures with bone defects who were treated using the Papineau technique at Cipto Mangunkusumo Hospital, Jakarta, Indonesia during the period of January 2017 to August 2019. Those with diabetes mellitus, severe liver disease, or malignancies were excluded. All surgical procedures were performed by one senior orthopedic surgeon.

**Results:**

A total of four subjects were enrolled in this study. All subjects were male, with a mean age of 29 ± 6.16 years of age. The mean time to granulation tissue was 21.5 ± 1.29 days, and the mean time to union was 6 ± 0 months. There were no complications.

**Conclusions:**

The Papineau technique may provide successful eradication of infection, reconstruction of bone defect, and soft-tissue closure. In addition, this technique is feasible and safe, and it could be performed in small healthcare centres.

## Highlights

• Cases of chronic osteomyelitis and infected nonunion after trauma remain a challenge.
• No consensus regarding the best method.
• In Indonesia studies regarding the use of the Papineau technique never been conducted.
• The Papineau technique could be performed in small healthcare centres.

## 1 Introduction

Despite advances in antibiotics and surgical techniques, cases of chronic osteomyelitis and infected nonunion after trauma remain a challenge to the orthopaedic field [1]. Extensive bone and soft tissue defects always accompany high-energy open fractures, and as a result the injured bones lose their ability to heal and are prone to infection [2]. Many techniques are used to treat these injuries, and there is currently no consensus regarding the best method [[3], [4], [5]]. Regardless of the method used, delayed union and nonunions are common [6] (see Fig. 3, Fig. 4).

In 1973, Papineau [7] introduced open bone grafting to treat these injuries, and such technique has been used for more than four decades with satisfactory results [8,9]. In this technique, serial local debridements are performed and followed by the excision of the infected bone and soft tissue necrotics [8,10,11]. Topical treatments are usually consisting of frequent moist dressing to promote granulation tissue or advanced wound care modalities and until granulation tissue is formed in the resected bone and soft tissue defect. Open autogenous cancellous bone grafting has been used to fil the resulting bone defect [12].

In the management of chronic osteomyelitis and infected nonunion of long bones of both lower and upper extremities, we aim to achieve the perfect control of infection and bony union. The Papineau technique is an alternative to treat such diseases in hospitals where microsurgery is not available, making closure of defect using flap is not feasible. In our country, studies regarding the use of the Papineau technique for treating chronic osteomyelitis and infected nonunion have never been conducted. We described our experience in treating patients with chronic osteomyelitis and infected non-union of tibial fractures using Papineau technique. This case series has been written according to PROCESS guideline [13].

## 2 Patients and methods

We retrospectively reviewed the records of patients with severe open tibial fractures with bone defects who were treated by our team between January 2017 and August 2019. The Papineau technique was used to treat four patients, all males aged 23–38 years.

Those with diabetes mellitus, severe liver disease, or malignancies were excluded. All patients provided written informed consent for all of the surgical procedures performed.

## 3 Surgery protocol

**Fig. 1:**
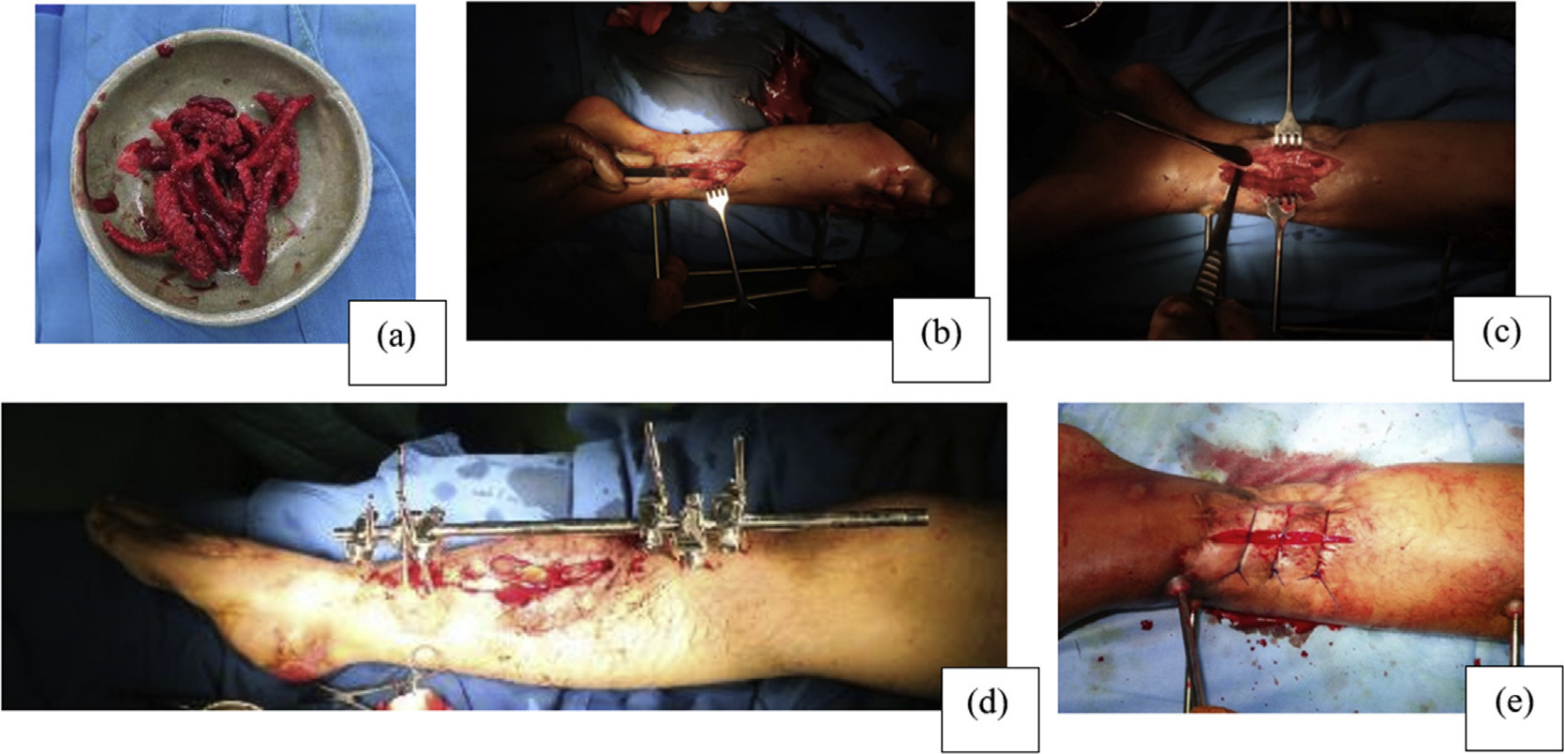
The surgical protocol. (a) Preparing iliac crest cancellous autograft. (b) Debridement, sequestrectomy, and decortication. (c) Bone graft insertion. (d) External fixator application. (e) Situational suture.

**Fig. 2:**
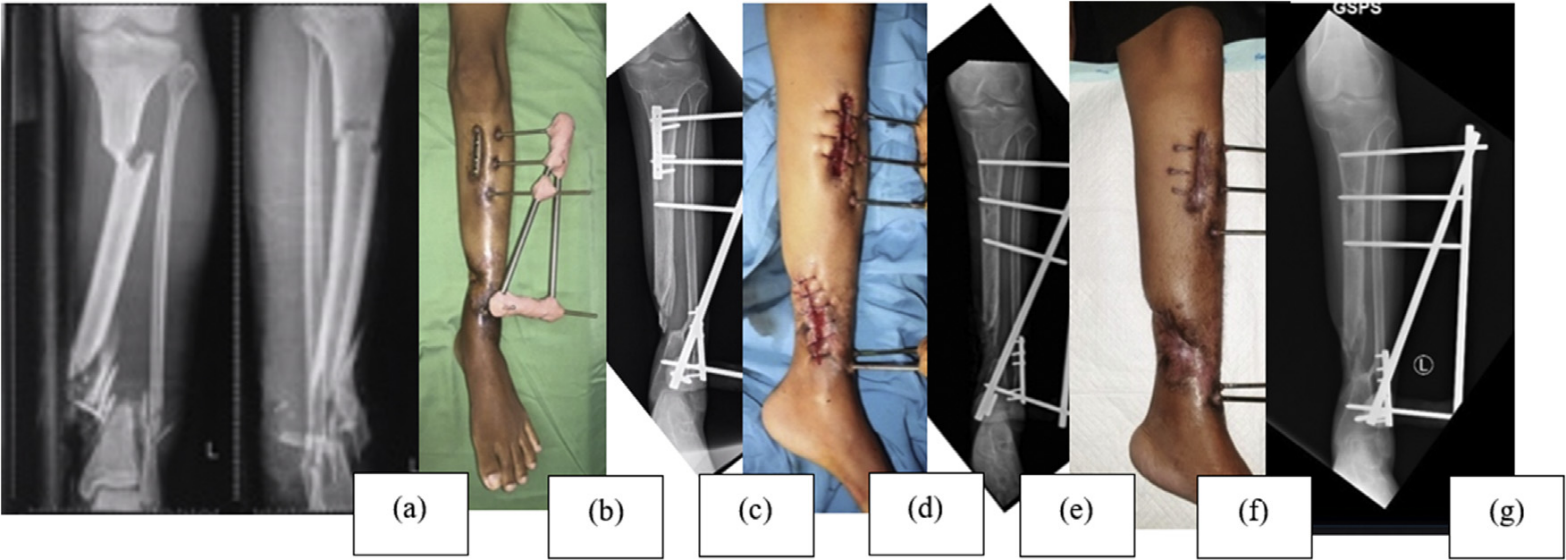
Case 1; a 23-year-old male with infected non-union of left distal tibial shaft. (a) Initial anteroposterior and lateral X-ray of the tibia. (b) Anterior preoperative view of the left leg – exposed sequester. (c) Preoperative anteroposterior X-ray of the left leg. (d) Anterior postoperative view of the leg. (e) Postoperative anteroposterior X-ray of the left leg. (f) Anterior view of the left leg, six months postoperatively – wound was healed. (g) Anteroposterior X-ray of the left leg, six months postoperatively – bridging bone formation.

All surgical procedures were performed by the same senior orthopedic surgeon. All patients received epidural anaesthesia. Debridement, including meticulous excision of local necrotic tissue, establishment of bone stability with single-sided external fixation with were performed on all patients. Haemostasis was achieved. Cefazolin was used for antibiotic prophylaxis at the time of the primary intervention after tissue samples were collected for culture and sensitivity examination, and antibiotics were adjusted based on culture results. The tibia was shortened until the edge of healthy and decortication around 1 cm from the fracture site. The gap was subsequently filled with sliced cancellous iliac bone autograft. Bone defects were packed circularly to cover all defect sides as well as both upper and lower fracture ends by 1 cm, (Fig. 1 and Fig. 2).

**Fig. 3:**
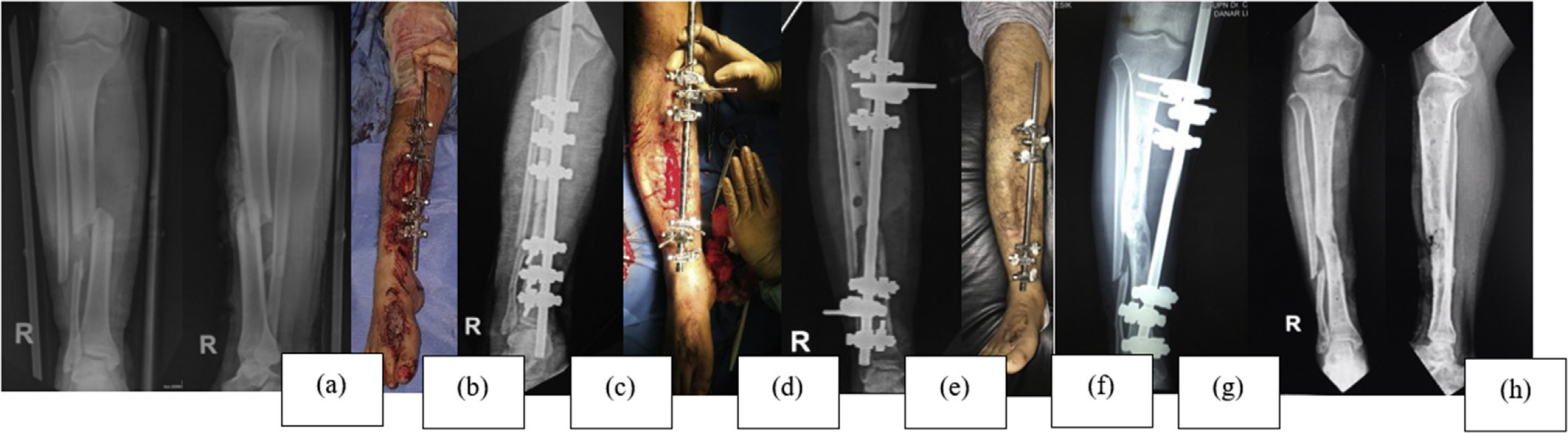
Case 2; a 24-year-old male with open fracture of the right tibial shaft. (a) Initial anteroposterior and lateral X-ray of the tibia. (b) Anterior preoperative view of the right leg. (c) Preoperative anteroposterior X-ray of the right leg. (d) Anterior postoperative view of the right leg – revision of external fixation more proximal and sliced cancellous bone graft. (e) Postoperative anteroposterior X-ray of the right leg. (f) Anterior view of the right leg, 13 months postoperatively – healed wound. (g) Anteroposterior X-ray of the right leg, 13 months postoperatively – bony union. (h) Post-implant removal

**Fig. 4:**
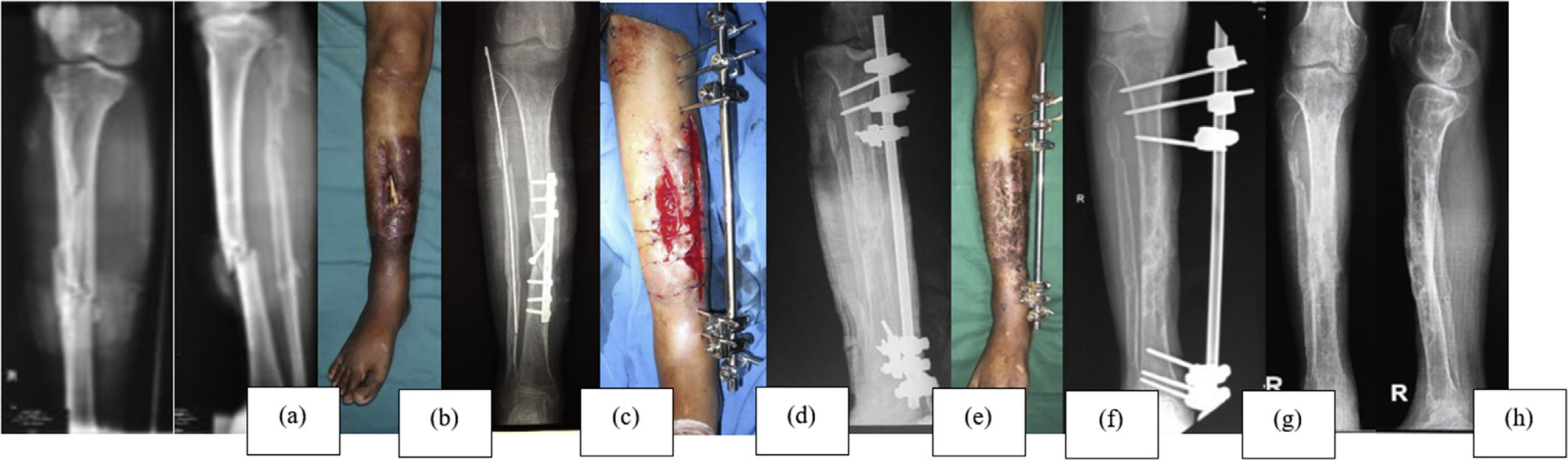
Case 3; a 38-year-old male with infected non-union of the right tibial shaft. (a) Initial anteroposterior and lateral X-ray of the tibia. (b) Anterior preoperative view of the right leg – exposed sequester. (c) Preoperative anteroposterior X-ray of the right leg. (d) Anterior postoperative view of the right leg – implant removal and external fixator application. (e) Postoperative anteroposterior X-ray of the right leg. (f) Anterior X-ray of the right leg, 9 months postoperatively – healed wound. (g) Anteroposterior view of the right leg, 9 months postoperatively – bony union. (h) Post-implant removal

## 4 Post-operative management

**Fig. 5:**
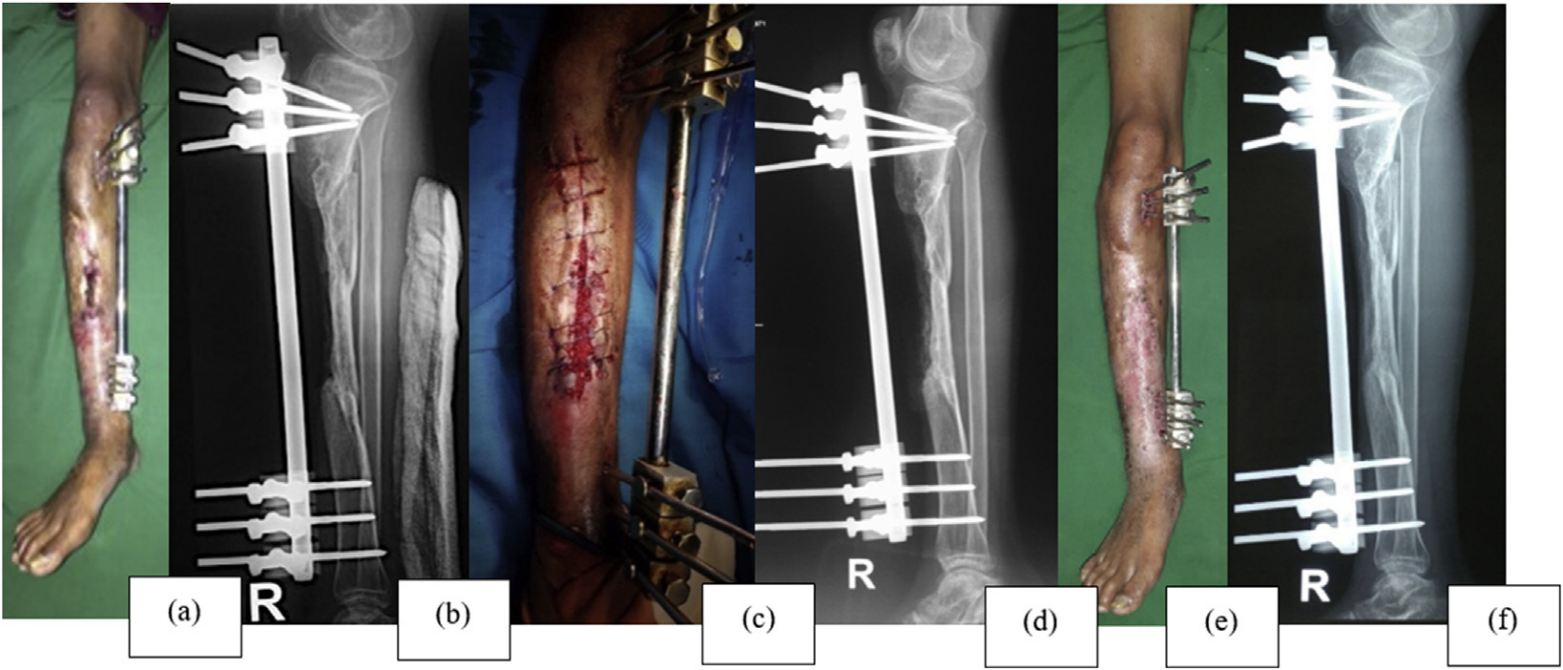
Case 4; a 27-year-old male with infected non-union of the right tibial shaft. (a) Anterior preoperative view of the right leg. (b) Preoperative lateral X-ray of the right leg. (c) Anterior view of the right leg. (d) Postoperative lateral X-ray of the right leg. (e) Anterior view of the right leg, 6 months postoperatively – healed wound. (f) Lateral X-ray of the right leg, 6 months postoperatively – bony union.

Antibiotics were administered based on bacterial culture results (see Fig. 5). Moist with honey dressings were changed twice daily and wound was evaluated once a week by the medical staff in the outpatient department. Partial weight-bearing was allowed when a continuous callus was observed on radiographs. External fixation was removed after a visible callus and sufficient stability and can bear body weight were achieved. Moreover, a brace was used until complete bone union had occurred.

## 5 Results

We included four subjects in this study. All subjects were male, with a mean age of 29 ± 6.16 years of age. The mean time to granulation tissue was 21.5 ± 1.29 days, and the mean time to union was 6 ± 0 months. No subjects developed complications. The median follow-up was 8.5 (6–13) months. Characteristics of the subjects are presented in Table 1, Table 2.

**Table 1:** Details of patients undergoing the technique.

Case number	Age (years)	Sex	Diagnosis	OTA classification	Time to granulation tissue coverage (days)	Time to wound healing (weeks)	Time to Union (months)	Length of follow-up (months)	Time to surgery	LLD (cm)	
										Preoperative	Postoperative
1	23	M	Infected non-union of left distal tibial shaft	42-C3	21	8	6	6	2 years	1	0
2	24	M	Open fracture of the right tibial shaft	42-A3	23	9	6	14	2 weeks	2	0.5
3	38	M	Infected non-union of the right tibial shaft	42-B3	22	8	6	9	1 year	0.5	0
4	27	M	Infected non-union of the right tibia lshaft	42-C2	20	8	6	8	3 years	1	0

M = male, LLD = leg length discrepancy.

**Table 2:** Details of the patients regarding previous surgeries.

Case number	Number of previous procedures	Previous procedures
1	1	Debridement, ORIF of the left fibula, external fixator application on the right tibia
2	1	Debridement and OREF of the right tibia
3	1	Debridement, ORIF of the right tibia-fibula
4	1	Debridement, OREF of the tibia

## 6 Discussion

Several approaches have been described to reduce significant soft tissue and bone defects, including open [7,8,14] or closed bone grafting [15], local or free muscle flap [16], and closed wound irrigation with suction [17]. The Papineau technique was developed to assist with the management of challenging bony defects and posttraumatic osteomyelitis. Papineau and others have reported high rates of success in eradicating chronic bone infection and addressing significant bone deficits [[7], [8], [9],^14^,^18^]. This technique involves thorough curettage or removal of the necrotic bone and unhealthy granulation in chronic osteomyelitis or infected nonunion of long bones followed by cortical and cancellous bone grafts and hematoma in recipient bed. Soft tissue cover age is not mandatory [1].

In this series, we found that all patients achieved union at a mean of 6 months. Moreover, there was no complication in our series. Bao et al. [2] treated 19 patients with open tibial fractures with soft tissue and segmental bone defects using Papineau technique combined with vacuum-assisted closure (VAC). Bone union was achieved in all patients at a mean of 33.88 ± 8.37 weeks (range, 25–33) weeks. No surgical complications occurred; however, one patient developed deep tissue infection. Karargyris et al. [19] treated seven patients (mean age, 32 years) with septic bone defects of the tibia with a Papineau technique and Ilizarov bone transport in a single-stage, followed by postoperative negative pressure wound dressing changes. All patients experience successful wound healing at a mean of 29 days. Six patients experienced successful bone regeneration and union at the docking side at a mean of 6 months. None experienced recurrence of infection. Kaushik et al. [1] treated 24 patients with chronic osteomyelitis of the tibia using modified a combination of Papineau technique and modern VAC system. The mean follow-up was 6 months (range, 5–24 months). They found that 80% of the cases achieved bony union and complete eradication of infection. Other studies have reported high rates of success for the treatment of chronic bone infection [20,21].

The three challenging problems with severe open tibial fractures are prevention of infection, bone union, and coverage of soft-tissue. Open bone grafting for the repair of infected bone defect can be divided into three stages: (1) complete debridement of all necrotic and infected bone and tissue; (2) cancellous bone grafting, and covered by the hematoma; (3) local wound care until coverage with granulation tissue followed by epithelization or skin grafting. Compared with other methods for repair of bone defects, such as vascularized bone graft, bone transport, and staged bone graft after wound closure, the Papineau technique simplifies repair by avoiding coverage of the wound by flaps (see Fig. 5).

Papineau technique provides adequate debridement and decortication to remove sequester and fibrotic tissue. Thus, it creates an environment for promoting bony union. Moreover, such method used sliced cancellous bone grafting which is arraged and merged with healthy hematoma. Bone grafting donor site must be preserved as it could be used repeatedly. Such technique also allows for adequate drainage by wound closure that is not tightly approximated. It also provides healthy granulation tissue formation and epitelization, which should be evaluated to determine the success of the Papineau technique [22]. Some surgeons have confirmed that the one-stage procedure can achieve satisfactory outcomes, including a shorter length of stay [23,24]. These findings emphasised a thorough debridement, as would be expected, since inadequate debridement increases the risk of recurrence of osteomyelitis and graft failure [25].

**Fig. 6:**
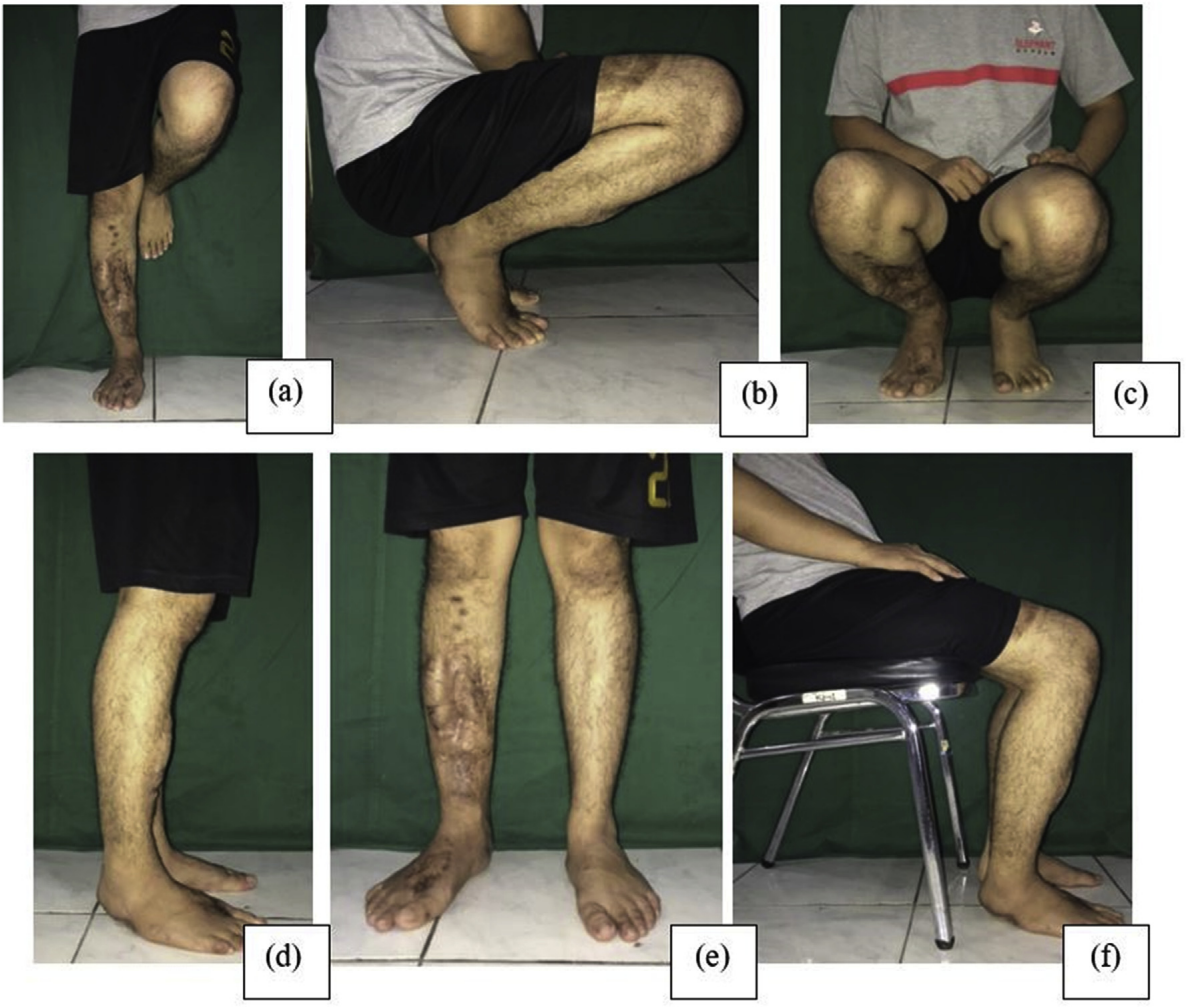
Photographs of case 2, obtained after 14 months of surgery. (a) Anteroposterior view of standing position with the affected leg, (b) lateral view of squatting position, (c) anteroposterior view of squatting position, (d) lateral view of standing position, (e) anteroposterior view of standing position, (f) lateral view of sitting position.

All patients had obtained full weight-bearing, normal range of motion, and returned to normal activity. Patients’ compliance and physiotherapy also play an important role in functional outcome (Fig. 6).

Our series demonstrated that Papineau technique is an effective and safe technique for treating bone defects of the tibia. However, as our study was limited by small number of subjects, further studies are required to investigate the safety and efficacy of Papineau technique for treating tibial bone defects. Moreover, such technique is limited by delayed wound healing, the necessity to use external fixator, and postoperative scar formation.

## 7 Conclusion

In conclusion, our series demonstrated that Papineau technique solely may provide successful eradication of infection, reconstruction of bone defect, and soft-tissue closure. Moreover, such technique is feasible and safe, and it could be performed in small healthcare centres in which facilities are limited. However, this technique has limitations including delayed wound healing, the necessity to use external fixator, and postoperative scar formation.

## Ethical approval

The ethical approval was not required for this case series.

## Sources of funding

The authors received no financial support for the research, authorship, and/or publication of this article.

## Author contribution

Bambang Gunawan: Concept of the study, data collection & interpretation, and writing the paper.

Mohammad Triadi Wijaya: Data collection, data interpretation and writing the paper.

Muhammad Alvin Shiddiqie Pohan: Data collection, data interpretation and writing the paper.

## Research registration number

This study has been registered at www.researchregistry.com, UIN no. researchregistry5089.

## Guarantor

Bambang Gunawan.

## Informed consent

Informed consent had been obtained from the patient before the manuscript was written.

## Provenance and peer review

Not commissioned, editor reviewed.

## Declaration of competing interest

The authors declare that there is no conflict of interest regarding the publication of this paper.
